# Estimation of Lewis Blood Group Status by Fluorescence Melting Curve Analysis in Simultaneous Genotyping of c.385A>T and Fusion Gene in *FUT2* and c.59T>G and c.314C>T in *FUT3*

**DOI:** 10.3390/diagnostics13050931

**Published:** 2023-03-01

**Authors:** Mikiko Soejima, Yoshiro Koda

**Affiliations:** Department of Forensic Medicine, Kurume University School of Medicine, Kurume 830-0011, Japan

**Keywords:** fluorescence melting curve analysis, fusion gene, *FUT2*, *FUT3*, Lewis blood group status, secretor status

## Abstract

Lewis blood group status is determined by two fucosyltransferase activities: those of *FUT2*-encoded fucosyltransferase (Se enzyme) and *FUT3*-encoded fucosyltransferase (Le enzyme). In Japanese populations, c.385A>T in *FUT2* and a fusion gene between *FUT2* and its pseudogene *SEC1P* are the cause of most Se enzyme-deficient alleles (*Se^w^* and *se^fus^*), and c.59T>G and c.314C>T in *FUT3* are tag SNPs for almost all nonfunctional *FUT3* alleles (*le^59^*, *le^59,508^*, *le^59,1067^*, and *le^202,314^*). In this study, we first conducted a single-probe fluorescence melting curve analysis (FMCA) to determine c.385A>T and *se^fus^* using a pair of primers that collectively amplify *FUT2*, se^fus^, and *SEC1P*. Then, to estimate Lewis blood group status, a triplex FMCA was performed with a c.385A>T and *se^fus^* assay system by adding primers and probes to detect c.59T>G and c.314C>T in *FUT3*. We also validated these methods by analyzing the genotypes of 96 selected Japanese people whose *FUT2* and *FUT3* genotypes were already determined. The single-probe FMCA was able to identify six genotype combinations: 385A/A, 385T/T, *se^fus^*/*se^fus^*, 385A/T, 385A/*se^fus^*, and 385T/*se^fus^*. In addition, the triplex FMCA successfully identified both *FUT2* and *FUT3* genotypes, although the resolutions of the analysis of c.385A>T and *se^fus^* were somewhat reduced compared to that of the analysis of *FUT2* alone. The estimation of the secretor status and Lewis blood group status using the form of FMCA used in this study may be useful for large-scale association studies in Japanese populations.

## 1. Introduction

The expression of Lewis blood group antigens, Lewis a (Le^a^) and Lewis b (Le^b^), is determined by the activity of *FUT2*-encoded fucosyltransferase (Se enzyme) and *FUT3*-encoded fucosyltransferase (Le enzyme) [1,2,3]. Secretors with at least one functional *FUT2* allele (*Se*) express soluble ABH(O) antigens in saliva and other secretions, while non-secretors, homozygotes for the nonfunctional *FUT2* (non-secretor) alleles (*se*), do not [3]. Weak secretors are homozygous for the weak-secretor allele (*Se^w^*) or compound heterozygous for *Se^w^/se* and are characterized by very low ABH antigen expression in secretions compared to secretors. This is because the activity of the Se enzyme encoded by *Se^w^* is very low but detectable due to a single nucleotide polymorphism (SNP), c.385A>T (p.Ile129Phe, rs1047781) [4,5]. In addition, Lewis-positive individuals with at least one functional *FUT3* allele (*Le*) have Le(a-b+) red cells in their secretors, Le(a+b-) in their non-secretors, and Le(a+b+) in their weak secretors. On the other hand, Lewis-negative individuals, homozygotes for the nonfunctional *FUT3* alleles (*le*), all have Le(a-b-) red cells [3]. Evidence is accumulating that secretor status and/or Lewis blood group status affects the susceptibility to a variety of clinical conditions, including some infectious diseases, inflammatory bowel disease, and plasma vitamin B12 levels [6,7,8,9,10,11,12,13,14,15].

**Figure 1:**
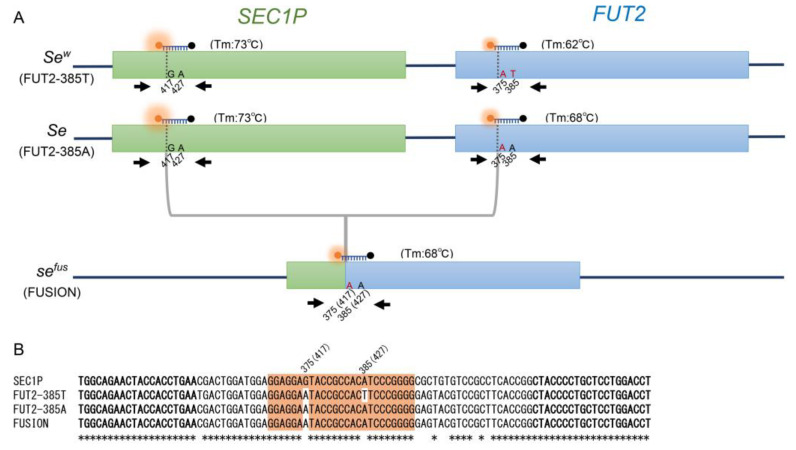
Genomic structure of *FUT2*, *SEC1P*, and *se^fus^*, and primer and probe positions used (**A**). The protein coding region of *FUT2* (FUT2-385A and FUT2-385T) is indicated by a blue box, that of *SECIP* is indicated by a green box, and that of *se^fus^* is indicated by green and blue boxes. The relative positions of the primers and probe anneal are indicated by black arrows, and the probe is indicated, in black combined with a fluorophore labeled at the 5′ end and a quencher labeled at the 3′ end. Nucleotides that differ from the sequence of the probe are indicated by red letters. Alignment of DNA sequences of amplified regions in FMCA; DNA sequences of *FUT2* (FUT2-385A: allele of A at rs1047781; FUT2-385T: allele of T at rs1047781; FUSION: *se^fus^* allele) and corresponding regions of *SEC1P*, are indicated (**B**). Forward primer and reverse primer sequences are shown in bold. The probe sequence is indicated by orange boxes and nucleotides that differ from the sequence of probe are uncolored.

To date, five *se* alleles resulting from non-allelic homologous recombination and several population-specific SNPs in *FUT2* and *FUT3* have been identified [16,17,18,19]. Among these, *se^fus^*, which results from an unequal crossover between *FUT2* and its pseudogene *SEC1P* (Figure 1A), is present almost exclusively in Japanese populations with a frequency of 5–9% [5,19]. *SEC1P* has high sequence similarity to *FUT2* and is located near *FUT2* on chromosome 19q13.3 [20,21]. In addition, the causal SNP for *Se^w^*, c.385A>T, is restricted to East and Southeast Asians, including Japanese people, with a frequency of about 50% [5,16,22,23]. Furthermore, three tag SNPs, c.59T>G (p.Leu20Arg, rs28362459), c.314C>T (p.Thr105Met, rs778986), and c.484A>G (p.Trp68Arg, rs28362463) in *FUT3*, were suggested to be useful for estimating *le* allele frequency in many populations [24]. In Japanese populations, since Se enzyme-deficient alleles other than *Se^w^* and *se^fus^* are quite rare and c.59T>G and c.314C>T are tag SNPs for almost all *le* alleles (*le^59^*, *le^59,508^*, *le^59,1067^*, and *le^202,314^*), determining these polymorphisms could provide an accurate estimate of secretor status and Lewis blood group status.

Fluorescence melting curve analysis (FMCA) is a simple, robust, and rapid closed-tube post-PCR method for detecting SNPs that analyzes the difference between the melting curve profiles of the fluorescence-labeled probe and PCR amplicon [25,26]. In addition, multiplex assays can be performed by using different fluorescent dyes [24,26]. Recently, we developed a triplex FMCA procedure for the genotyping of three tag SNPs, c.59T>G, c.314C>T, and c.484A>G, in *FUT3* to estimate *le* allele frequency [24].

In this study, we performed a FMCA that simultaneously determined the frequency of c.385A>T and *se^fus^* using a single probe. A triplex FMCA was then performed to estimate Lewis blood group status in Japanese people, adding primers and probes to detect c.59T>G and c.314C>T of *FUT3* to c.385A>T and *se^fus^* assay system.

## 2. Materials and Methods

### 2.1. DNA Samples

The genomic DNAs of 96 Japanese people from Fukuoka whose *FUT2* haplotypes had been determined previously using established methods such as the allele specific PCR and/or DNA sequencing [5] were used in this study. The study protocol was reviewed and approved by the ethical committee of Kurume University (approval no. 22158).

### 2.2. Asymmetric PCR for c.385A>T and se^fus^ of FUT2

The nucleotide positions of the *FUT2* and *SEC1P* genes were numbered as described previously [21]. All primers and probes were synthesized by Eurofins Genomics K.K (Tokyo, Japan). As shown in Figure 1A, we used a pair of primers that collectively amplify *FUT2*, *se^fus^*, and *SEC1P*. The forward primer, 5′-TGGCAGAACTACCACCTGAA-3′, matches exactly 337–356 bp of *FUT2* and 379–398 bp of *SEC1P*, and is the same primer that previously detected c.385A>T by an unlabeled probe HRM analysis [27]. The reverse primer, 5′-AGGTCCAGGAGCAGGGGTAG-3ʹ, matches exactly the reverse sequences of 414–433 bp of *FUT2* and of 456–475 bp of *SEC1P*, and is the same primer that previously detected *se^fus^* by a TaqMan probe assay and HRM analysis [19,28]. The SEC1P-FUT2 probe, HEX-5′-GGAGGAGTACCGCCACATCCCGGGG-3′-black hole quencher 1, matches exactly 411–435 bp of *SEC1P*, and 369–393 bp of *FUT2*, but differs by one base from the wild-type (385A allele) and *se^fus^*, and differs by two bases from the 385T allele. The asymmetric PCR reaction mixture with a final volume of 10 µL contained 5 µL of *TaKaRa Taq* HS Perfect Mix containing modified *Taq* DNA polymerase, which has neither 5′-3′ exonuclease nor 3′-5′ exonuclease activities (Takara, Tokyo, Japan), 50 nM of the forward primer, 500 nM of the reverse primer, 200 nM of the SEC1P-FUT2 probe, and 2–20 ng of genomic DNA. The PCR was conducted on LightCycler 480 Instrument II (Roche Diagnostics, Tokyo, Japan) with the following thermal conditions: 45 cycles of denaturation at 95 °C for 5 s, and annealing/extension at 60 °C for 15 s.

### 2.3. Triplex PCR for c.385A>T and se^fus^ of FUT2, c.59T>G and c.314C>T of FUT3

Recently, we conducted a triplex FMCA for the genotyping of three tag SNPs, c.59T>G, c.314C>T, and c.484A>G, in *FUT3* to estimate *le* allele frequency [24]. However, c.484A>G is highly specific to African populations and has not yet been observed in Asian populations, including Japanese populations. Therefore, in this study, previously reported primers and probes were added to the c.385A>T and *se^fus^* of the *FUT2* assay system to detect c.59T>G and c.314C>T, excluding c.484A>G. However, since a HEX-labeled probe was used to detectc.385A>T and *se^fus^*, a FAM-labeled probe was used instead of a HEX-labeled probe to detect c.59T>G in this study. The primer and probe concentrations for c.59T>G and c.314C>T were as follows: 50 nM of each forward primer, 250 nM of each reverse primer, 50 nM of a probe for c.59T>G, and 100 nM of a probe for c.314C>T. The primer and probe concentrations for c.385A>T and *se^fus^* and the thermal conditions of the asymmetric PCR were the same as described above.

### 2.4. FMCA for Detection of c.385A>T and se^fus^ of FUT2, c.59T>G and c.314C>T of FUT3

The PCR products were then heated to 95 °C for 1 min and cooled to 40 °C for 1 min, and fluorescence data were acquired using the VIC/HEX/Yellow 555 filter (excitation–emission: 533–580 nm) and/or the FAM filter (465–510 nm) and/or the Cy5/Cy5.5 filter (618–660 nm) during heating from 50 to 80 °C at a 0.1 °C/s ramp rate. Melting curve genotyping and melting temperature (Tm) analyses were carried out using the LightCycler 480 gene scanning software.

## 3. Results

### 3.1. FMCA for Detection of c.385A>T and se^fus^ of FUT2

**Figure 2:**
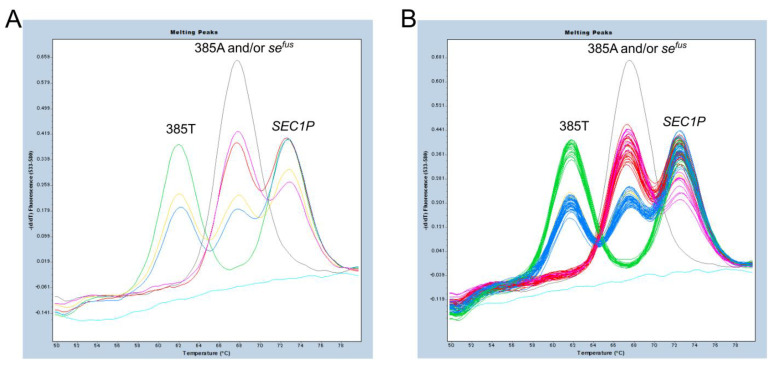
Melting peak profiles of FMCA for detection of c.385A>T and *se^fus^*. Six Japanese (**A**) and 96 Japanese subjects (**B**) whose *FUT2* genotypes were already determined were selected. The subjects with genotypes 385A/A (red), 385T/T (green), *se^fus^*/*se^fus^* (gray), 385A/T (blue), 385A/*se^fus^* (pink), and 385T/*se^fus^* (yellow) were clearly identified. The negative control is shown in light blue.

In this study, we first attempted to detect c.385A>T and *se^fus^* with a single probe using a primer set that collectively amplified 97 bp amplicons of *FUT2*, *se^fus^*, and *SEC1P*. As shown in Figure 1B and Figure 2A, the highest Tm value around 73 °C was observed for the *SEC1P* amplicon because the probe sequence exactly matches that of *SEC1P*. On the other hand, an intermediate Tm value around 68 °C was observed for the 385A allele amplicon because the probe sequence differed by one base, and the lowest Tm value around 62 °C was observed for the 385T allele amplicon because the probe sequence differed by two bases. In addition, in *se^fus^*, the nucleotides corresponding to the positions of 375 bp and 385 bp in *FUT2* (or 419 bp and 429 bp in *SEC1P*) were both “A”, and therefore, the Tm value for the *se^fus^* amplicon was the same (around 68 °C) as that for the 385A amplicon (Figure 1A,B).

A chromosome with the 385A or 385T allele had two regions (413–437 bp of *SEC1P* and 369–393 bp of *FUT2*) that hybridized with the probe (Figure 1A,B). On the other hand, a chromosome with the *se^fus^* allele had only one region that hybridized with the probe because *se^fus^* had been generated by an unequal crossover between the 253 and 416 bp positions of *SEC1P* and between the 211 and 374 bp positions of *FUT2*, and the position of 385 bp of *FUT2* is located immediately at the 3′ region of the recombination sequence [19].

**Figure 3:**
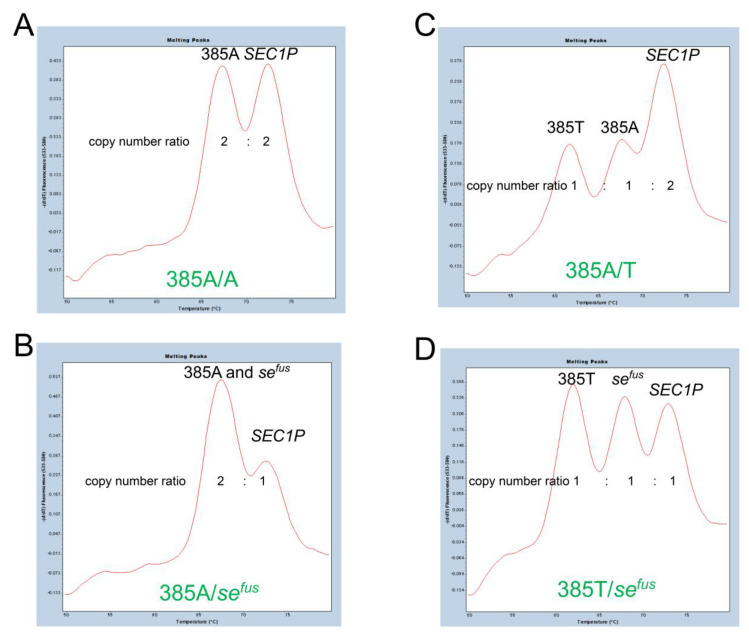
Melting peak profiles of FMCA for four selected subjects with or without *se^fus^*. Melting peak profiles of 385A/A (**A**), 385A/*se^fus^* (**B**), 385A/T (**C**), and 385T/*se^fus^* (**D**). The copy number ratio of each peak is indicated.

We then analyzed 96 Japanese people whose *FUT2* haplotypes had already been determined by allele-specific PCR and/or DNA sequencing [5]. Homozygotes of *se^fus^* (*se^fus^*/*se^fus^*) showing only one melting peak at around 68 °C, and homozygotes of 385T allele (385T/T) showing two melting peaks at around 73 °C and 62 °C were completely separated by the default settings of the LightCycler 480 gene scanning software (normal sensitivity, score threshold 0.70, resolution threshold 0.10). On the other hand, homozygotes of the 385A allele (385A/A) and heterozygotes of 385A/*se^fus^* with two melting peaks at around 73 °C and 68 °C, or heterozygotes of c.385A>T (385A/T) and heterozygotes of 385T/*se^fus^* with three melting peaks at around 73 °C, 68 °C, and 62 °C, were classified into the same group in the default settings. However, it was possible to separate 385A/A from 385A/*se^fus^* and 385A/T from 385T/*se^fus^* by changing the settings to normal sensitivity with a score threshold of 0.85 and a resolution threshold of 0.00 (Figure 2B). The reason for this is that the peak height corresponding to *SEC1P* for *se^fus^* heterozygotes (385A/*se^fus^* and 385T/*se^fus^*, Figure 3B**,**D) are relatively lower than that corresponding to *SEC1P* for subjects without *se^fus^* (Figure 3A**,**C).

Although one weak secretor with the genotype *Se^w^*/*se^628^*, determined by a Sanger sequencing analysis, was misclassified as *Se/Se^w^* in this FMCA because the nucleotide at position 385 of *se^628^* was an “A”, the other results were completely in accordance with previous ones [5]. The *FUT2* genotyping results of conducting a FMCA of 96 Japanese people were as follows: 23 were 385A/A, 38 were 385A/T, 22 were 385T/T, 9 were 385A/*se^fus^*, 2 were 385A/*se^fus^*, and one was *se^fus^*/*se^fus^*. In addition, the repeatability the results was confirmed because the results of two independent assays were identical.

### 3.2. Triplex FMCA for Detection of c.385A>T and se^fus^ of FUT2, c.59T>G and c.314C>T of FUT3

**Figure 4:**
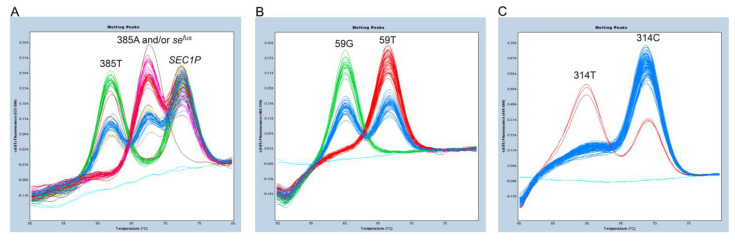
Melting peak profiles of triplex FMCA of 96 Japanese subjects. (**A**) Results for detection of c.385A>T and *se^fus^* of *FUT2*. The subjects with genotypes between 385A/A and 385A/*se^fus^* and between 385A/T and 385T/*se^fus^* were not completely separated automatically. The unknown group is indicated in brown. Light blue indicates the negative control. (**B**) Results of detection of c.59T>G of *FUT3*. The subjects with genotypes of 59T/T are shown in red, those with genotypes of 59T/G in blue, those with genotypes of 59G/G in green. The negative control is shown in light blue. (**C**) Results for detection of c.314C>T of *FUT3*. The subjects with genotypes of 314C/C are shown in blue, those with genotypes of 314C/T in red. The negative control is shown in light blue.

We then attempted a triplex FMCA that could estimate the Lewis blood group status of the Japanese people by adding primers and probes that detect c.59T>G and c.314C>T in *FUT3* and c.385A>T and adding the *se^fus^* assay system. The melting curve genotyping results for c.385A>T and *se^fus^* from the triplex FMCA were similar to those from the single-probe FMCA with the default settings; however, unlike the single-probe FMCA, some melting peaks were divided into unknown groups (Figure 4A, shown in brown) when the score threshold was increased. Therefore, automatic discrimination by a software is difficult, but it seems possible to separate them by manual visual discrimination. This was possible because, as described above, the peak height corresponding to *SEC1P* in *se^fus^* heterozygotes was relatively lower than the peak height corresponding to *SEC1P* in subjects without *se^fus^*. On the other hand, c.59T>G and c.314C>T were clearly separated automatically, as described previously (Figure 4B**,**C). The *FUT3* genotyping results of conducting a FMCA of 96 Japanese people were as follows: 35 were 59T/T, 43 were 59T/G, and 18 were 59G/G, while 93 were 314C/C and three were 314C/T.

Table 1 shows the *FUT2* and *FUT3* genotypes, secretor status, and Lewis blood group status of the 96 Japanese subjects estimated by the present triplex FMCA. Thus, by the FMCA, 60 of the 96 Japanese subjects were estimated to be Lewis-positive secretors with a Lewis phenotype of Le(a-b+); 16 were Lewis-positive weak secretors of Le(a+b+), one was a Lewis-positive non-secretor of Le(a+b-), and 19 were Lewis-negative subjects of Le(a-b-). In addition, 11 of the 19 people with a phenotype of Le(a-b-) were estimated to be secretors and 8 were estimated to be weak secretors.. Incidentally, conventional serological Lewis phenotyping is somewhat difficult because it depends largely on the strength and specificity of the anti-Le^a^ and anti-Le^b^ antibodies used and the skill of the observer [29]. In fact, in a previous study in which we analyzed the *FUT2* of the same subjects used in this study, we misdiagnosed the serological Lewis phenotype Le(a+b+) as Le(a+b-) [5]. This may have been due to the specificity of the anti-Le^b^ antibody used. Thus, we classified Le(a+b-) subjects as being of the Se enzyme-deficient phenotype, which includes both weak secretors and non-secretors. In any case, with the exception of one subject (as mentioned above), the previous Lewis phenotyping results were also compatible with the estimate of the Lewis phenotype made by the FMCA.

## 4. Discussion

Several real-time PCR based methods were developed to identify c.385A>T or *se^fus^* individually, including high-resolution melting (HRM) analysis and a TaqMan (hydrolysis probe) assay [19,27,28,30]. In this study, we developed an FMCA method to detect c.385A>T and *se^fus^* by an asymmetric PCR using a single probe. The probe-based FMCA showed significant Tm change (about 5–6 °C) between a wild type (385A) allele and/or *se^fus^*, and between a mutant type (385T) allele and *SEC1P*. Thus, this single-probe assay accurately determined 385A>T substitution. *se^fus^* was found almost solely in the Japanese population with a frequency of 5–9% [19], and thus, 11 of the 96 Japanese subjects were *se^fus^* heterozygotes (nine subjects were 385A/*se^fus^*, two subjects were 385T/*se^fus^*) in this study. As described previously, an artificial recombinant of *SEC1P* and *FUT2* was generated during PCR amplification when a relatively small fragment specific to the *se^fus^* sequence was amplified [28]. To avoid the production of an artificial recombinant of *SEC1P* and *FUT2*, we selected primers that had amplified *se^fus^* and *SEC1P* in the previous studies [19,28], and that amplified *se^fus^*, *SEC1P*, and *FUT2* in the present study. The present method has an advantage over the previous methods, which could only detect c.385A>T or *se^fus^*, in that it can simultaneously detect *se^fus^* and the c.385A>T of *FUT2* in a single assay.

Because the *se^fus^* allele contains the 3′ region of the wild-type *FUT2* sequence, it was previously misidentified as a functional 385A allele when c.385A>T was genotyped using primers that specifically amplified the *FUT2* sequence surrounding 385A>T [19,28]. In the present study, in fact, the Tm value of the *se^fus^* signal was also the same as that of the 385A allele signal, but since a chromosome with *se^fus^* lacks *SEC1P*, it appeared that the zygosity of *se^fus^* could be determined by the peak height of the *SEC1P* signal. Namely, *se^fus^*/*se^fus^* lacked the *SEC1P* signal and *se^fus^* heterozygotes had relatively lower peak *SEC1P* signal height. The relatively lower peak *SEC1P* signal height of in the *se^fus^* heterozygotes could not be detected by the software of the real-time PCR instrument used with the default settings for melting curve genotyping. According to the instrument manual (LightCycler 480 Instrument Operator’s Manual), ‘score’ is an index of the similarity between the melting curves of a sample and the melting curve of the standard that is most similar to the sample, and ‘resolution’ is the index of the dissimilarity between the melting curve of the sample and the melting curve of the second most similar standard. Therefore, subtle differences in the relatively lower peak *SEC1P* signal height in the *se^fus^* heterozygotes could have been detected by increasing the score threshold and decreasing the resolution threshold from the default settings.

In addition, we developed a triplex FMCA prodcedure that could simultaneously detect the c.385A>T and *se^fus^* of *FUT2* and the c.59T>G and c.314C>T of *FUT3*. Because the resolution was somewhat lower for the triplex FMCA than for the single-probe FMCA, it was difficult to automatically separate the six genotype combinations using the software. Nevertheless, it was possible to first separate them into four groups, *se^fus^*/*se^fus^*, 385T/T, 385A/A plus 385A/*se^fus^*, and 385A/T plus 385T/*se^fus^*, by the default settings, and then 385A/A was further separated from 385A/*se^fus^* and 385A/T from 385T/*se^fus^* by manually observing the relative peak *SEC1P* signal heights. Therefore, we could estimate not only secretor status but also Lewis blood group status in a single assay.

The c.375A>G (synonymous SNP, rs1800026) of *FUT2* has been observed in African and Oceanian populations [31,32]. The *FUT2* allele with this SNP would be determined as *SEC1P* in the present method. However, in other populations, including the Japanese population, this SNP seems to be rarely observed. Therefore, such a misdiagnosis is unlikely to occur in the genotyping of Japanese subjects. A limitation of the present method is that it cannot detect rare known *se* alleles such as *se^571^* and *se^628^* and rare unknown *se* and *le* alleles. In fact, we misdiagnosed one *se^628^* allele as a functional allele by the present method. In addition, this method is useful almost exclusively for Japanese populations.

Sanger sequencing, the golden standard for the determination of SNPs, can detect these rare *se* alleles. However, compared to Sanger sequencing, for the whole coding region of *FUT2*, the present FMCA method is simple, cost-effective, and rapid, making it suitable for high-throughput analysis [33]. In addition, it is impossible to detect *se* alleles generated by non-allelic homologous recombination such as *se^fus^* by Sanger sequencing for the whole coding region of *FUT2*. Therefore, the probe-based FMCA procedure used in this study may have some advantages over Sanger sequencing.

The association between phenotypic polymorphisms of blood types, as represented by the ABO blood group, and specific diseases has been analyzed in the past by case-control studies, but few reports have shown an association of those with specific symptoms such as the presence of duodenal ulcers [34]. On the other hand, recent large-scale analyses using genomic DNA, including genome-wide association studies (GWAS), have revealed associations between SNPs in blood group genes and various unexpected diseases. Typical examples include the association of the ABO blood group gene polymorphisms with pancreatic cancer and thromboembolic and arterial disease [35,36]. Although the mechanisms by which blood group polymorphisms are involved in the pathogenesis of each of these diseases remain to be elucidated, the identification of associations with known genetic polymorphisms that seem to be unrelated to disease is one of the advantages of large-scale GWAS. As further analyses are conducted, it is possible that more diseases or clinical conditions will be found to be associated with secretor status and Lewis blood group.

Furthermore, while serological Lewis phenotyping could not determine the secretory status of Lewis-negative subjects, i.e., those without Le(a-b-), *FUT2* genotyping could determine the secretory status of Lewis-negative subjects. A recent study suggested that red cells with Lewis a phenotype displayed strongly reduced SARS-CoV2-susceptibility [37]. However, as mentioned above, serological Lewis phenotyping is somewhat difficult. Thus, the estimation of Lewis phenotypes by reliable *FUT2* and *FUT3* genotyping is a useful alternative method for phenotyping, and the FMCA method used here appears to be a valid and feasible method for large-scale association studies of both secretor status and Lewis blood group status in Japanese populations.

## Figures and Tables

**Table 1:** *FUT2* and *FUT3* genotypes and secretors and Lewis blood group phenotypes estimated by the triplex FMCA.

c.385A>T	Fusion Gene	*FUT2* Genotype	Secretor Phenotype	c.59T>G	c.314C>T	*FUT3* Genotype	Lewis Phenotype	Number
A/A	-	*Se/Se*	Secretor	T/T	C/C	*Le*/*Le*	Le(a-b+)	11
					C/T	*Le*/*le^202,314^*	Le(a-b+)	2
				T/G	C/C	*Le*/*le^59^*	Le(a-b+)	7
					C/T	*le^59^*/*le^202,314^*	Le(a-b-)	1
				G/G	C/C	*le^59^*/*le^59^*	Le(a-b-)	3
A/T	-	*Se/Se^w^*	Secretor	T/T	C/C	*Le*/*Le*	Le(a-b+)	13
				T/G	C/C	*Le*/*le^59^*	Le(a-b+)	19 *
				G/G	C/C	*le^59^*/*le^59^*	Le(a-b-)	6
T/T	-	*Se^w^/Se^w^*	Weak secretor	T/T	C/C	*Le*/*Le*	Le(a+b+)	9
				T/G	C/C	*Le*/*le^59^*	Le(a+b+)	5
				G/G	C/C	*le^59^*/*le^59^*	Le(a-b-)	8
A/A	one copy	*Se/se^fus^*	Secretor	T/T	C/C	*Le*/*Le*	Le(a-b+)	2
				T/G	C/C	*Le*/*le^59^*	Le(a-b+)	6
				G/G	C/C	*le^59^*/*le^59^*	Le(a-b-)	1
T/T	one copy	*Se^w^/se^fus^*	Weak secretor	T/G	C/C	*Le*/*le^59^*	Le(a+b+)	2
A/A	two copies	*se^fus^/se^fus^*	Non-secretor	T/G	C/C	*Le*/*le^59^*	Le(a+b-)	1

* One weak secretor with genotypes *Se^w^*/*se^628^*, estimated by Sanger sequencing analysis, was misdiagnosed as *Se/Se^w^* by this FMCA because the nucleotide at position 385 of *se^628^* is an “A”; *le^59^* includes *le^59^*, *le^59,^*^508^, and *le^59,1067^*.

## Data Availability

The data presented in this study are available on request from the corresponding author.
